# Prevalence of Temporomandibular Disorders in Completely Edentulous Patients Attending the Dental Consultation and Treatment Center in Casablanca: A Descriptive Cross-Sectional Study

**DOI:** 10.7759/cureus.83398

**Published:** 2025-05-03

**Authors:** El Mehdi Jouhadi, Ichraq Benazouz, Maya Nabli, Mouna Hamza, Zineb Al Jalil, Khalid El Boussiri

**Affiliations:** 1 Department of Fixed Prosthodontics, Faculty of Dental Medicine, Hassan II University of Casablanca, Casablanca, MAR; 2 Department of Biology and Basic Subjects, Faculty of Dental Medicine, Hassan II University of Casablanca, Casablanca, MAR; 3 Department of Pediatric Dentistry, Faculty of Dental Medicine, Hassan II University of Casablanca, Casablanca, MAR; 4 Laboratory of Community Health Epidemiology and Biostatistics, Faculty of Dental Medicine, Hassan II University of Casablanca, Casablanca, MAR; 5 Department of Dentistry and Biology and Basic Subjects, Faculty of Dental Medicine, Hassan II University of Casablanca, Casablanca, MAR

**Keywords:** complete dentures, edentulous, mouth, prevalence, temporomandibular disorders

## Abstract

Introduction: Temporomandibular disorders pose complex challenges to efficient dental diagnosis and follow-up care. These disorders can significantly impact the physiological activities of edentulous patients, contributing to their high prevalence. This pioneering study aimed to determine the prevalence of temporomandibular disorders among edentulous patients seeking treatment at the Prosthodontic Department of the Dental Consultation and Treatment Center in Casablanca.

Methods: Over an eight-month period, all patients meeting the inclusion criteria and consulting the Prosthodontic Department were invited to participate anonymously in the study. Evaluation of the signs and symptoms of temporomandibular disorders was performed using the Helkimo index. The outcomes were analyzed by correlating the research variables and the prevalence of the signs and symptoms of temporomandibular disorders through the chi-squared test using IBM SPSS Statistics for Windows, Version 20.0 (Released 2011; IBM Corp., Armonk, New York, United States).

Results: A total of 130 patients were included in this investigation. Our findings revealed that the predominant age group within our study population comprised individuals over 65 years, accounting for 40.8% of the participants. Age was observed to influence the expression of temporomandibular dysfunction symptoms. Among the population reporting temporomandibular disorders, patients with recent complete dentures appeared to be the most affected (29.23%). The selected index for diagnosing these disorders demonstrated satisfactory results in terms of the alignment between the anamnestic and clinical indices, along with consistency in findings.

Conclusion: Our study revealed a notable prevalence of temporomandibular disorders in these patients, indicating the need for future research on management strategies for these disorders, especially for those experiencing severe symptoms.

## Introduction

The temporomandibular joint (TMJ) is a vital component of the stomatognathic system, comprising various internal and external structures that enable complex movements [1]. However, this system may undergo alterations over an extended period, often resulting in the gradual onset of temporomandibular disorder (TMD) symptoms [2].

TMDs refer to a range of conditions characterized by pain or dysfunction in the TMJ and/or muscles of mastication. In recent decades, researchers have focused on the etiology and diagnostics of TMDs [3]. The etiology of TMD is complex and multifactorial, potentially involving joint hyperlaxity and joint hypermobility, psychological factors (stress, depression, anxiety, and personality), hormonal factors (estrogen), history of microtrauma (on the head or neck), behavioral factors (parafunctions, grinding, clenching, and abnormal head posture), social factors (which can affect pain perception and response to pain), and occlusal factors [4].

Among these, occlusion remains one of the most controversial etiological factors in TMD. Some authors argue that occlusion has minimal impact on the etiology of TMD [5]. In contrast, others affirm a strong correlation between occlusal dysfunctions and the onset of TMD, emphasizing the importance of identifying pathogenic occlusal dysfunctions that may exacerbate TMD symptoms [6,7]. According to Pullinger and Seligman, occlusal dysfunctions could act as co-factors in TMD etiology rather than as initiating factors [8]. In addition to occlusion, several studies have investigated the correlation between tooth loss and TMD. Malheiros et al. reported a direct relationship between tooth loss and TMD symptoms, with the severity of TMD being significantly higher in edentulous patients [9]. These findings are consistent with previous studies, which have concluded that TMD symptoms are more prevalent among complete denture wearers compared to individuals with natural dentition [10,11]. Predisposing factors include changes in the mandible's rest position, a reduction in the vertical dimension of occlusion, and the positional drifting of the condyles in the mandibular fossae [12].

The study aims to determine the prevalence of TMD in a sample of completely edentulous Moroccan patients who visit the Prosthodontic Department of the Dental Consultation and Treatment Center in Casablanca (CCTD) and to analyze associations with demographic and prosthetic variables, in order to facilitate the diagnosis of these disorders and their delicate management.

## Materials and methods

A descriptive cross-sectional study was conducted over eight months, from September 2020 to April 2021, at the Prosthodontic Department of the CCTD in Casablanca, Morocco. All eligible patients consulting during this period were invited to participate anonymously in the survey. Ethical approval for this study was obtained from the Pedagogic and Research Conduct Committee of the Faculty of Dental Medicine, Hassan II University of Casablanca (approval number: FMDC-pr2c/06-2020). The study included all participants who met the following inclusion criteria: completely edentulous patients without complete dentures, who visited the Prosthodontic Department at the CCTD, patients wearing old complete dentures seeking renewal of their prosthesis, and newly fitted bimaxillary edentulous patients treated at the Prosthodontic Department.

Upon confirming the eligibility of each participant, informed consent was obtained. Data were collected by a university-based clinician (J.E.L.), an expert in occlusion and TMDs, and a dental intern (M.N.), who received prior calibration. A theoretical and practical training session was carried out before data collection. All clinical and anamnestic data were then collected collaboratively, under the direct supervision of the senior expert examiner, to ensure consistency and minimize interobserver variability.

The evaluations were conducted in two stages: In the first step, a questionnaire (see Appendices) was used to gather sociodemographic information, including age, gender, level of education, general health, and dental history (tooth loss and prosthetic history). Additionally, the Helkimo Anamnestic Index (Ai) was applied to assess anamnestic dysfunction through a yes/no questionnaire [13,14]. The responses allowed for the classification of patients into the following categories: Patients who answered "no" to all questions were categorized as Ai 0, indicating that they were free of any TMD symptoms. Patients who answered "yes" to any of the questions 1, 2, or 4 were classified as Ai I, which reflects the presence of one or more mild symptoms, such as TMJ sounds (clicking, popping, or grating), jaw fatigue, or jaw stiffness during slow movements or upon waking. In contrast, patients who answered "yes" to any of the questions 3, 5, 6, 7, or 8 were classified as Ai II, indicating the presence of one or more severe symptoms (limited jaw movement, jaw locking, mandible dislocation and its painful movement, and painful TMJ region and/or masticatory muscles).

In the second step, an intra- and extra-oral clinical examination of the masticatory system was carried out following the guidelines of the Helkimo Clinical Dysfunction Index (Di). This index evaluates visible dysfunctions in the stomatognathic system by analyzing the clinical signs of TMD [13,14]. The clinical evaluation included the assessment of maximum mouth opening, TMJ dysfunction (palpation of interarticular space to detect tenderness or pain and auscultation of the TMJ to assess the presence of joint sounds such as clicking or crepitus), myalgia (pain in the masticatory muscles), TMJ pain tenderness, and pain during mandible movement. The severity of clinical signs was scored on a three-point scale: 0 points indicated the absence of symptoms, 1 point represented mild symptoms, and 5 points denoted severe symptoms. Each individual's total dysfunction score ranged from 0 to 25 points, with higher scores indicating acute or serious dysfunction. 

Statistical analysis was carried out in collaboration with the Department of Epidemiology and Biostatistics using IBM SPSS Statistics for Windows, Version 20.0 (Released 2011; IBM Corp., Armonk, New York, United States). Qualitative variables were reported as frequencies and percentages, and the chi-squared (χ²) test was used to assess associations, with the significance level set at p<0.05. The analysis focused on the association between TMD symptoms and sociodemographic characteristics, as well as the distribution of TMD symptoms within the study sample according to the Helkimo Ai.

## Results

Among the 130 patients included in this study, 57.69% were men and 42.31% were women with 40.77% aged over 65 years. Table 1 shows the sociodemographic variables, while Table 2 illustrates the association with the presence of TMD symptoms.

**Table 1:** Sociodemographic profile of the included patients (n=130)

Characteristics		n (%)
Age (years)	Less than 35	1 (0.77%)
	35-45	4 (3.07%)
	45-55	28 (21.54%)
	55-65	44 (33.85%)
	Over 65	53 (40.77%)
Gender	Male	75 (57.69%)
	Female	55 (42.31%)
Education level	No education	57 (43.85%)
	Primary school	58 (44.61%)
	Secondary school	11 (8.46%)
	University	4 (3.08%)
Socioeconomic level	Low	108 (83.08%)
	Middle	22 (16.92%)
	High	0 (0%)

**Table 2:** Analysis of the association between sociodemographic variables and TMD symptoms TMD: temporomandibular disorder

Characteristics		Absence of TMD symptoms (%)	Presence of TMD (%)
Age (years)	Less than 35	0.77%	0%
	35-45	1.54%	1.54%
	45-55	1.54%	20%
	55-65	17.69%	16.15%
	Over 65	16.15%	24.61%
Gender	Male	20.77%	36.92%
	Female	16.92%	25.38%
Education level	No education	14.62%	29.23%
	Primary school	18.46%	26.15%
	Secondary school	4.62%	3.85%
	University	0%	3.08%
Socioeconomic level	Low	33.08%	50%
	Middle	4.62%	12.31%
	High	0%	0%

Concerning edentulousness, 55.38% of patients were edentulous for less than five years, followed by 28.46% of the patients who were edentulous for 5-10 years. There was no significant association between the duration of edentulousness and the presence of TMDs. Upon reviewing the prosthetic history, it was observed that out of 130 patients, 58 (44.62%) presented with outdated dentures. A correlation was found between the origin of denture fabrication, the duration of prosthesis wear, and the incidence of TMDs.

Patients whose prostheses were designed by non-professionals (individuals who are not formally trained or accredited in dentistry) were more likely to develop TMDs, a difference that was statistically significant (p=0.029). Additionally, the longer a patient wore their complete bimaxillary dentures (CBD), the less likely they were to experience TMDs, with a statistically significant difference (p=0.017). These variables are summarized in Table 3. This highlights a statistically significant inverse correlation between the duration of CBD wear and the incidence of TMDs (p=0.017).

**Table 3:** The association between prosthetic history and clinical index TMD: temporomandibular disorders; CBD: complete bimaxillary denture; CCTD: Dental Consultation and Treatment Center in Casablanca

Characteristics		Absence of TMDs (%)	Presence of TMDs (%)
Place of denture design (χ2=8.985; p=0.029)	Non-professionals	7.69%	19.23%
	Private doctor	0%	6.15%
	Prosthodontic Department at CCTD	3.85%	7.69%
Prosthetic status (χ2=13.758; p=0.017)	Has never worn a denture	26.15%	29.23%
	CBD dating less than 5 years	3.85%	18.46%
	CBD dating 5-10 years	6.15%	11.54%
	CBD dating 10-15 years	0%	1.54%
	CBD dating 15-20 years	0%	1.54%
	CBD dating more than 20 years	1.54%	0%

The Helkimo Ai was recorded in all patients in the study. Out of the 130 patients, 46.92% reported no TMD and were classified as Ai 0. Additionally, 23.85% of the patients reported moderate TMD (Ai I), and 29.23% exhibited severe TMD (Ai II). Based on the results from the correlation of the Ai with the clinical index, a clear association emerges between the Ai and the presence of TMD. The analysis indicates a significance level set at 0 (Table 4).

**Table 4:** Distribution of TMD symptoms according to the Helkimo Ai among study participants χ2=22.395; p=0.000 (significant) TMD: temporomandibular disorder; Ai: Anamnestic Index

Characteristics		Frequency, n (%)
Ai 0	No symptoms	61 (46.92%)
Ai I	TMDs of moderate severity	31 (23.85%)
Ai II	Patients with severe TMD	38 (29.23%)

Finally, the Di was used to categorize our patients into four groups. A total of 37.7% of the patients scored 0 points (Di 0), indicating a clinical absence of dysfunction symptoms. In the Di I group, 55.38% of patients scored between 1 and 4 points, reflecting mild symptoms of dysfunction. The Di II group included 5.38% of patients who scored between 5 and 9 points, corresponding to moderate dysfunction symptoms. Finally, 1.54% of patients were classified into the Di III group, having scored between 10 and 25 points, denoting severe symptoms of dysfunction. Notably, a proportion of patients classified as Ai 0 also presented with mild or even moderate clinical signs of dysfunction, according to the Di scores. This reflects the known variability between subjective symptom reporting and objective clinical findings in TMD assessment.

## Discussion

Based on this study's results, older patients exhibited a higher susceptibility to TMDs, with a statistically significant difference (p=0.002). This observation contrasts with the findings of other authors, who have reported that symptoms indicative of TMD tend to be more prevalent among individuals aged 35-50 years, with a notable decline observed in older age groups (65-75 years) [15]. It is conceivable that as individuals age, TMJs undergo degenerative changes, impacting the ability to repair and remodel adequately in response to heightened functional demands, ultimately leading to dysfunction [16,17]. 

Regarding the association between gender and the prevalence of TMD, both men and women were similarly affected. These results align with previous studies [18]. However, other authors reported that women are more affected than men. For instance, Zissis et al. found that women manifest significantly more frequent TMD signs and symptoms than men (65.7%), a predominance that may be attributed to hormonal changes in the bone structures of post-menopausal women [19]. Other research supports this theory [20,21].

As highlighted in other studies, education level is strongly associated with TMD [22,23]. In fact, 43.8% of our patients were illiterate. We can assume that patients with advanced education would be more aware of the importance of preserving their teeth through proper dental hygiene and regular dental visits.

In terms of edentulousness, our findings are consistent with those reported by Zakir et al. in 2020 [24], where 59% of their patients had been edentulous for less than five years and 29% for 5-10 years. Other authors, however, show different proportions; they suggest that most cases of edentulousness are less than five years old [25]. Our results report no association between the history of edentulousness and the presence of TMDs. However, the origin of prosthesis emerged as a statistically significant variable related to the expression of TMD symptoms. It is noteworthy that non-professional practitioners showed less efficiency than private dentists in prosthesis fabrication and adjustment. This may be explained by a lack of knowledge among unqualified practitioners regarding the principles of designing total bimaxillary prostheses, particularly in maintaining the vertical dimension of occlusion and achieving occlusal balance. A previous study confirmed this hypothesis, demonstrating that the vertical dimension of old dentures was too low in 61 patients, six of whom exhibited signs and symptoms of TMD. The severity of TMD signs and symptoms in these patients decreased from three to five weeks after correcting the occlusion of their old dentures [26]. Additionally, it has been demonstrated that patients with poorly fitting and functioning dentures experience more frequent TMD symptoms [27]. Therefore, it is crucial to create and maintain properly adjusted complete dentures, which involves recording an accurate centric relation, establishing an appropriate vertical dimension of occlusion, achieving bilateral balanced occlusion, and performing regular follow-ups. These steps help facilitate the adaptation of the masticatory system and prevent the resumption of muscle, bone, and joint remodeling, which could lead to an acute phase of TMD.

In this study, we selected the Helkimo index for its effectiveness as an epidemiological instrument, designed to investigate the prevalence of TMDs and assess the treatment needs based on the severity of findings [3]. Indeed, our results indicate a perfect correlation between the Ai and the presence of TMDs. The analysis shows a significance level of 0. This suggests that the Ai could be equally effective in diagnosing TMDs in edentulous patients, comparable to the clinical index, which is often considered more precise due to its objective nature, free from the subjective experiences of patients, who might tend to exaggerate their symptoms.

Limitations of the study

This study encountered significant challenges due to COVID-19 pandemic-related disruptions at CCTD, which led to a marked decline in patient volume over the eight-month study period. As a result, only 130 participants were enrolled. Future research should incorporate robust sample size calculations to more accurately assess the prevalence and severity of TMDs in the Moroccan population. Additionally, this study may not fully represent the Moroccan population, as patients from higher socioeconomic backgrounds who often seek private rather than public healthcare for denture renewals were not adequately represented among those consulting at CCTD, introducing potential selection bias. Another limitation is the absence of multivariate analysis, which could have controlled for confounding factors and strengthened the validity of the findings. This aspect could be further developed in future studies.

## Conclusions

The diagnosis of TMD can be effectively achieved through both anamnestic and clinical clues, requiring detailed questioning to determine the underlying causes. Our results indicate that elderly, completely edentulous patients with recent, poorly designed prostheses are at higher risk of developing dysfunctional symptoms. It is imperative for dental care providers to understand the anatomical and physiological consequences of complete denture wear on the TMJ. This study revealed a significant prevalence of TMDs among these patients, highlighting the need for future work on the effective management strategies of these disorders, particularly in patients experiencing severe symptoms.

## Appendices

Questionnaire

This questionnaire has been developed as part of a research study entitled "Prevalence of Temporomandibular Disorders in Completely Edentulous Patients Attending the Dental Consultation and Treatment Center in Casablanca: A Descriptive Cross-Sectional Study". The purpose of this study is purely academic and aims to contribute to the understanding of temporomandibular disorders in edentulous individuals. Participation is entirely voluntary, and all responses will be treated with strict confidentiality and anonymity.

By completing this questionnaire, the participant confirms their voluntary participation and provides informed consent to be included in this study. Please kindly complete Sections A, B, and C.1. Section C.2 is reserved for clinical use and must be completed by the examining clinician.

Section A: Sociodemographic Profile

1. Age: *Only one answer is valid*

Less than 35 

35 to 45

45 to 55

55 to 65

Over 65

2. Sex: *Only one answer is valid*

Male

Female

3. Education level: *Only one answer is valid*

No education

Primary school

Secondary school

University

4. Socioeconomic level: *Only one answer is valid*

Low

Middle

High

5. Socioprofessional category: *Only one answer is valid*

Without profession

Daily or temporary worker

Farmer

Artisan

Cadre and senior official

Large merchant

Dentist, doctor, veterinarian ...

Other:

Section B: Medical Anamnesis

1.  General health status: *Several responses are possible*

Cardiovascular diseases

Diabetes (type 1 or type 2)

Respiratory diseases

Neurological disorders

Psychiatric conditions (depression, anxiety disorders)

Rheumatologic diseases (rheumatoid arthritis, lupus)

Gastrointestinal diseases (GERD, Crohn's disease)

Endocrine disorders (thyroid dysfunction)

History of malignancy

Immunosuppressive conditions

Other:

2.  Cause of teeth loss:* Several responses are possible*

Caries

Periodontal pathology

Traumatic pathology

3.  Edentulous status: *Only one answer is valid*

Less than 5 years

Between 5 years and 10 years

More than 10 years

4.  Prosthetic status: *Only one answer is valid*

Has never worn a denture

Complete bimaxillary dentures dating less than 5 years

Complete bimaxillary dentures dating 5 to 10 years

Complete bimaxillary dentures dating 10 to 15 years

Complete bimaxillary dentures dating 15 to 20 years

Complete bimaxillary dentures dating more than 20 years

5.  Place of denture design: *Only one answer is valid*

Non-professionals

Private doctor

Prosthodontic Department at the Dental Consultation and Treatment Center in Casablanca (CCTD)

Section C: Evaluation of Temporomandibular Disorders

1.  Evaluation of subjective symptoms using the Helkimo Anamnestic Index (Ai)

Several responses are possible.

**Table 5:** Evaluation of subjective symptoms using the Helkimo Anamnestic Index (Ai) TMJ: temporomandibular joint

Symptoms		
Do you have a sound (clicking or crepitation) in the area of TMJ?	Yes	No
Do you have jaw rigidity during awakening or mandible movement?	Yes	No
Do you have fatigue in the jaw area?	Yes	No
Do you have difficulty when opening the jaw?	Yes	No
Do you have locked mandible while opening the mouth?	Yes	No
Do you have pain in the TMJ or in masticatory muscles?	Yes	No
Do you have pain during movement of the mandible?	Yes	No
Do you have luxation of the mandible?	Yes	No

2.  Clinical examination criteria according to the Helkimo Clinical Dysfunction Index (Di)

This question is reserved for clinical use and must be completed by the examining clinician.

**Table 6:** Clinical examination criteria according to the Helkimo Clinical Dysfunction Index (Di) TMJ: temporomandibular joint

Symptom	Criteria	Score
Range of mandibular opening	Normal range of movement	0
	Slightly impaired mobility	1
	Severe impaired mobility	2
Impaired TMJ function	Smooth movement without joint sounds and deviation ≤2 mm	0
	Joint sounds in one or both joints and deviation ≥2 mm on opening or closing	1
	Locking or luxation of the joint	2
Muscle pain	No tenderness to palpation	0
	Tenderness to palpation in 1-3 sites	1
	Tenderness to palpation in 4 or more sites	2
TMJ pain	No tenderness to palpation	0
	Tenderness to palpation in 1-3 sites	1
	Tenderness to palpation in 4 or more sites	2
Pain on movement of the mandible	No pain on movements	0
	Pain on 1 movement	1
	Pain on 2 or more movements	2

Each individual's total dysfunction score ranged from 0 to 25 points: 0 points indicated the absence of clinical symptoms (Di 0), 1-4 points represented mild dysfunction (Di I), 5-9 points indicated moderate dysfunction (Di II), and 10-25 points corresponded to severe or acute dysfunction (Di III).
